# Locomotor Trajectories of Stroke Patients during Oriented Gait and Turning

**DOI:** 10.1371/journal.pone.0149757

**Published:** 2016-02-19

**Authors:** Céline Bonnyaud, Nicolas Roche, Angele Van Hamme, Djamel Bensmail, Didier Pradon

**Affiliations:** 1 Inserm Unit 1179, Team 3: Technologies and Innovative Therapies Applied to Neuromuscular diseases, UVSQ, APHP Service de physiologie et d’exploration fonctionnelle, Hôpital Raymond Poincaré, 92380, Garches, France; 2 Inserm Unit 1179, Team 3: Technologies and Innovative Therapies Applied to Neuromuscular diseases, UVSQ, APHP Service de Médecine Physique et Réadaptation, Hôpital Raymond Poincaré, 92380, Garches, France; INSERM U894, FRANCE

## Abstract

**Background:**

The Timed Up and Go (TUG) test is widely used to assess locomotion in patients with stroke and is considered to predict the risk of falls. The analysis of locomotor trajectories during the TUG appears pertinent in stroke patients. The aims of this study were i) to analyze locomotor trajectories in patients with stroke during the walking and turning sub-tasks of the TUG, and to compare them with healthy subjects, ii) to determine whether trajectory parameters provide additional information to that provided by the conventional measure (performance time), iii) to compare the trajectory parameters of fallers and non-fallers with stroke and of patients with right and left hemisphere stroke, and iv) to evaluate correlations between trajectory parameters and Berg Balance Scale scores.

**Methods:**

29 patients with stroke (mean age 54.2±12.2 years, 18 men, 8 fallers) and 25 healthy subjects (mean age 51.6±8.7 years, 11 men) underwent three-dimensional analysis of the TUG. The trajectory of the center of mass was analyzed by calculation of the global trajectory length, Hausdorff distance and Dynamic Time Warping. The parameters were compared with a reference trajectory during the total task and each sub-task (Go, Turn, Return) of the TUG.

**Results:**

Values of trajectory parameters were significantly higher for the stroke group during the total TUG and the Go and Turn sub-tasks (p<0.05). Moreover, logistic regression indicated that these parameters better discriminated stroke patients and healthy subjects than the conventional timed performance during the Go sub-task. In addition, fallers were distinguished by higher Dynamic Time Warping during the Go (p<0.05). There were no differences between patients with right and left hemisphere stroke.

**Discussion and Conclusion:**

The trajectories of the stroke patients were longer and more deviated during the turn and the preceding phase. Trajectory parameters provided additional information to timed performance of this locomotor task. Focusing rehabilitation programs on lead-up to turn and turning could be relevant for stroke patients since the Turn was related to the balance and the phase preceding the turn seemed to distinguish fallers.

## Introduction

Stroke is a major cause of disability in adults [1]. It frequently results in hemiparesis (partial paralysis of one side of the body) which causes slow gait with kinematic anomalies [2],[3]. Methods of quantitative gait analysis are becoming increasingly used in clinical practice to aid clinical decision-making by the assessment of spatio-temporal, kinematic and kinetic parameters [4]. Three-dimensional analysis is the current gold standard for the biomechanical assessment of patients with abnormal gait [5]. This typically involves the analysis of straight-line gait, however straight-line gait does not reflect daily life situations which include curved paths, obstacle circumvention and U-turns [6]. Curved paths and obstacle circumvention have been studied in healthy subjects [7],[8],[9] and more recently in subjects with stroke [10],[11],[12]. The Timed Up and Go (TUG) test [13],[14] involves rising from a chair, walking 3m, turning 180°, returning, and sitting down again. It thus reflects the main aspects of gait required in daily life. It is rated according to performance time [13],[14],[15]. The test is useful and is quick and easy to perform, therefore it is widely used in clinical practice for the assessment of global locomotor capacity in stroke patients. However, performance time does not provide any information regarding the biomechanical behaviour of patients during the test. Moreover, several authors have recommended refining the TUG test by timing each sub-task (23), as well as carrying out a biomechanical analysis of each sub-task (24).

A recent approach to the analysis of biomechanical behavior during tasks involving curved gait is the study of trajectory. Locomotor trajectory has been evaluated in healthy subjects during imposed straight and curved walking (indicated by a line drawn on the floor) [7] as well as walking through doors with different spatial orientations [16]. The results suggest that the control of the locomotor pattern is based on the whole-body locomotor trajectory, rather than a sequence of foot pointings. To our knowledge, only one study has investigated locomotor trajectory in stroke patients [17]. The trajectories of patients with stroke and healthy subjects were evaluated in a virtual environment which created 5 different scenes of translational optic flow (a pattern of apparent motion of objects, surfaces, and edges in a visual scene caused by the relative motion between an observer and the scene) [17]. The medio-lateral and antero-posterior trajectories of the center of mass (COM) were computed while subjects were instructed to “walk straight with respect to the scene they were visualizing”. Displacement of the COM was altered in the patients with motor disorders in contrast with the healthy subjects who displayed stereotypical behavior. The authors suggested that this was the result of an alteration in perception and/or a poor integration of sensorimotor information. No studies have analyzed the spontaneous trajectories of patients with stroke in a “real environment” during tasks encountered in daily life. Since many stroke patients have spatial disorders, such an analysis would be clinically relevant to guide rehabilitation, and the TUG test appears to be a pertinent test on which to base the analysis. Moreover, this test can easily be broken down into sub-tasks to analyze different locomotor task. In addition, it has been shown that perception of body verticality is altered following right hemisphere stroke [18], thus locomotor trajectories may differ between patients with right and left hemisphere stroke.

Several methods in the literature have been used to evaluate locomotor trajectories. The amount of deviation from either a required or an averaged trajectory appears to be particularly relevant [7],[16]. Trajectory deviation can be quantified using several parameters. The simplest is the Euclidean distance, however this method is not sufficiently accurate to compare groups with different gait velocities [19]. The Hausdorff Distance (HD) and Dynamic Time Warping (DTW) appear to be appropriate for the present study since these parameters can be used to compare the geometry and the spatio-temporal time series of two sequences of different lengths. HD and DTW have been used to evaluate moving objects [20], for handwriting recognition [21] and to study walking behavior [22],[23]. Since the gait of stroke patients is slower than that of healthy subjects, these parameters are pertinent [19],[20] to compare their locomotor trajectories.

The TUG test is considered to indicate a risk of falls [24],[25]. Older subjects are classified as fallers if they take 13.5sec or more to perform the test and stroke patients are considered at risk of falls if they take 15sec or more [24],[25]. However, a more recent study has suggested this test is not sufficiently accurate to discriminate fallers and non-fallers [26]. We thus propose to use HD and DTW to determine whether these trajectory-related parameters might permit to distinguish stroke-related fallers and non-fallers.

The aims of this study were thus: i) to analyze locomotor trajectories using HD and DTW in patients with stroke during the walking and turning sub-tasks of the TUG and to compare them with healthy subjects; ii) to determine whether trajectory parameters provide additional information to that of the conventional measure (performance time); iii) to compare the trajectory parameters of fallers and non-fallers with stroke and of patients with right and left hemisphere stroke and iv) to evaluate correlations between trajectory parameters and Berg Balance Scale scores. This study is the first to assess the locomotor trajectories of patients with stroke in real life conditions. The results should yield pertinent information for clinicians, helping to orientate rehabilitation and perhaps also to identify potential fallers. We hypothesized: 1) that the trajectories of stroke patients would deviate from those of healthy subjects, particularly during the Turn sub-task of the TUG since this task is the most challenging regarding stability, 2) that trajectory parameters would provide additional information to performance time, 3) that trajectories would differ between fallers and non-fallers and that since right hemisphere large vessel distribution stroke may alter perception of body verticality, it may also alter the locomotor trajectories and 4) that longer trajectories would be related to a poorer BBS scores since we supposed that patients with impaired balance would deviate from the optimal trajectory to ensure stability.

## Methods

### Subjects

Twenty nine patients with chronic stroke (mean age 54.2±12.2 years, 18 men), who were in- or outpatients in our department of physical medicine and rehabilitation, and twenty five healthy subjects (mean age 51.6±8.7 years, 11 men) were included. This number of subjects was sufficient to obtain a minimum statistical power of 95% with a significance level (alpha error) of 0.05, based on calculation of the effect size and statistical power using previous data published on TUG performance in stroke subjects [14],[27] [28]. Based on the current sample size and the results of DTW during the Turn and trajectory length, the effect sizes obtained were respectively 1.56 and 2.37 and the subsequent powers were respectively 0.99 and close to 1 which allow us to be confident in our results. Inclusion criteria were: hemiparesis following stroke, over 18 years old and able to carry out the TUG test several times consecutively without using an assistive device. Exclusion criteria were the diagnosis of other neurological or orthopedic conditions, or having undergone surgical procedures during the last 6 months. Participants’ characteristics are presented in Table 1. Patients were considered as fallers if they had fallen at least once within the last 3 months. The fallers’ characteristics are presented in Table 2. Eight patients had gait-related falls and constituted the group of fallers in this study. Six of these patients had fallen indoors (one while walking, one while walking in a narrow space, three while turning and one tripped on a rug) and 2 patients had fallen outdoors in crowded spaces. Six patients were not included in the faller group since they fell in conditions that did not involve walking (in the bathtub, on the stairs, rising from a chair, crossing an obstacle and entering a car). All patients were found to be capable of providing informed consent during the medical examination, and all gave written informed consent in accordance with the ethical codes of the World Medical Association. The study was approved by our local ethics committee (Comité de protection des personnes Ile de France XI, Ref 13005. CNIL, Ref DR-2013-283).

**Table 1:** Subject characteristics.

	Stroke patients (n = 29)	Healthy subjects (n = 25)
Age (years)	54.2±12.2	51.6±8.7
Height (m)	1.68±0.09	1.67±0.1
Weight (kg)	73.2±16.2	65.6±14.7
Gender (m/f)	18m / 11w	11m / 14w
Mean self-selected gait speeds for the walking phases of the TUG (m/s)	0.4±0.006	0.7±0.04
Time since stroke (years)	7.9±5.7	-
Stroke etiology	19 ischemia / 10 hemorrhage	-
Hemiparetic side	12 right / 17 left	-
Falls	8 fallers related to gait	-
Modified Ashworth sum	4 [2;7]	-
MRC sum	23 [19;25]	-
Foot sensation	1 [1;2]	-
Toe proprioception	2 [1;3]	-
Barthel index	100 [95;100]	-
NFAC	7 [7;7]	-
BBS	51 [49;52]	-
ABC	76,3±12,9	-

Patients with stroke had a significantly decreased gait speed compared to healthy subjects (p<0.05)

Falls: patients were considered as fallers if they had fallen at least once within last 3 months

Spasticity: median [interquartile range Q1;Q3] of the sum of quadriceps, rectus femoris, hamstring and triceps surae spasticity assessed with Modified Ashworth Scale (0–4).

MRC (Medical Research Council scale): median [interquartile range Q1;Q3] of the sum of hip, knee and ankle flexor and extensor strength (0–5)

Foot sensation: median [interquartile range Q1;Q3] of the foot sensation score assessed with the Nottingham Sensory Assessment (0 = absent, 1 = impaired, 2 = normal)

Toe proprioception: median [interquartile range Q1;Q3] of the toe proprioception score assessed with the Nottingham Sensory Assessment (0 = absent, 1 = direction incorrect, 2 = direction ok, inaccurate position, 3 = direction ok, position accurate to 10°)

Barthel index: median [interquartile range Q1;Q3] Barthel score (0 to 100)

NFAC: median [interquartile range Q1;Q3] New Functional Ambulation Classification score (0 to 8)

BBS: median [interquartile range Q1;Q3] Berg Balance Scale score (0 to 56)

ABC: mean±sd Activities-specific Balance Confidence scale (0 to 100%)

**Table 2:** Characteristics of the fallers and non-fallers.

	Fallers (n = 8)	Non-fallers (n = 21)
Age (years)	59,5±11,6	52,2±12,1
Gender (m/f)	3m / 5w	15m / 6w
Hemiparetic side	2 right / 6 left	10 right / 11 left
TUG (sec)	19,7±1,8	19,1±4,9

### Experimental procedure

All participants performed 3 TUG tests under standardized conditions. They wore the same type of comfortable shoes [29], sat on a stool set to 100% of the distance from the head of the fibula to the floor [30] with their knees flexed to 100°, their feet placed symmetrically and their arms held out from the body [31],[32],[33]. Participants were instructed to rise from the stool, walk 3m, turn around a cone towards their paretic side (non-dominant side for healthy subjects), return to the stool and sit down, at their own comfortable speed. The TUG tests were recorded with a motion analysis system (Motion Analysis Corporation, Santa Rosa, CA, USA, sampling frequency 100 Hz). Thirty-four markers were fixed, by the same person, to specific bony landmarks according to the Helen Hayes marker set [34],[35],[5]. The marker set was used to create a 12-segment rigid-link model of the body using Dempster's anthropometric table which is routinely used in gait analysis [36],[37]. Markers were tracked by 8 infrared cameras and trajectories were filtered using a low-pass Butterworth filter with a cut off frequency of 6 Hz [38]. An open-source Biomechanical Tool Kit package for MATLAB [39] was used to define the phases of the gait cycle and sub-tasks of the TUG. The gait phases were defined according to Perry [3] and sub-tasks of the TUG were defined according to previous studies [33],[40],[41]. The three sub-tasks of the TUG that involve walking were analysed: the first oriented-gait sub-task (Go) which begins at toe off of the first step and ends with the first foot strike in the direction of the turn, the turning sub-task (Turn) which ends at the first foot strike lined up with the stool and the second oriented-gait sub-task (Return) ends with foot strike of the last step prior to the turn to sit [12].

Locomotor trajectory was evaluated by the displacement of the center of mass (COM) with the following equation 1:

$$COMx=\frac{m_{1}\;x_{1}+m_{2}\;x_{2}+{\ldots ..+m}_{i}\;x_{i}}{M}=\frac{1}{M}\sum_{i=1}^{N}m_{i}\;x_{i}$$

where M = whole body mass

mi = mass of the ith segment = (whole body mass) x (mass fraction for ith segment from the anthropometrics.dat file)

xi = the x-coordinate of the center of mass for the ith segment with respect to the calibration origin

N = the number of body segments

The parameters analyzed were:

➢ time to perform the Go, Turn and Return sub-tasks of the TUG, and total TUG time
➢ length of the COM trajectory, HD and DTW

The trajectories of each patient and healthy subject were compared with the reference trajectory, defined as the mean of the healthy subjects’ trajectories which were time-resampled [16].

*Trajectory length* was calculated with the following equation 2

$$\mathrm{Trajectory length}=\sum \sqrt{(x_{i+1}}-x_{i}{)}^{2}+{\left(y_{i+1}-y_{i}\right)}^{2}$$

**Fig 1:**
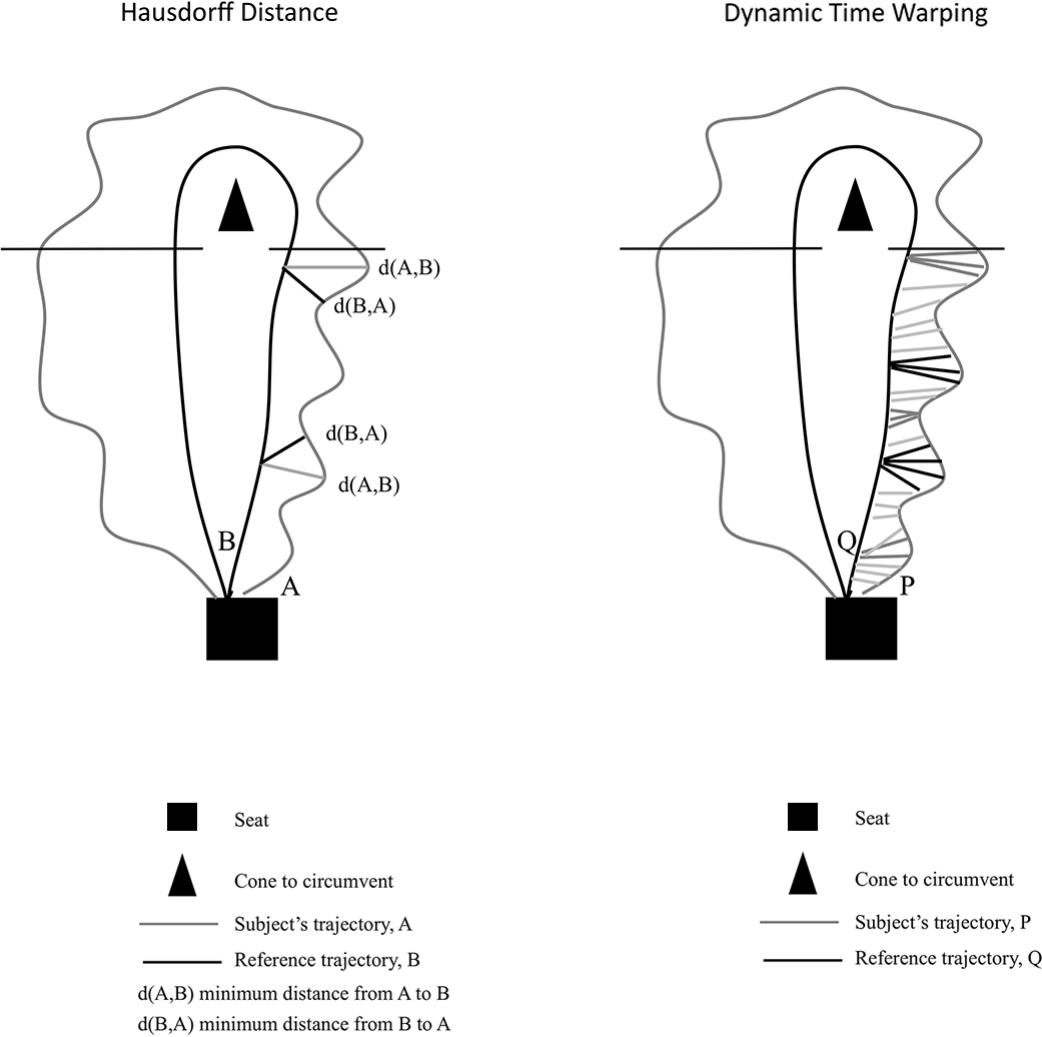
Explication of Hausdorff distance and dynamic time warping between a subject’s trajectory and the reference trajectory for a TUG sub-task.

*HD* corresponds to the geometric analysis of the trajectory. Each point of the considered subject’s trajectory is assigned to the closest point of the reference trajectory and conversely, each point of the reference trajectory is assigned to the closest point of the considered subject’s trajectory (Fig 1). HD is the greatest of all the distances from a point in one set (A) to the closest point in the other set (B). HD is thus sensitive to corner points.

HD was calculated with the following equation 3.

HD(A,B)=max{d(A,B),d(B,A)}

where d(A,B) and d(B,A) are the direct (minimum) Euclidean distances between two sets, A and B [23].

The result is in cm. The greater the distance, the higher the deviation from the reference trajectory.

*DTW* is a spatio-temporal analysis which corresponds to the path of cumulative distances that minimize the warping cost (pair of matching points) of two time series, P and Q [42]. The algorithm first calculates the distance between each point of the subject’s trajectory and reference trajectory and then searches an optimal matching (minimal cost) between sequence points (a point of a sequence is associated with one or more points of the other sequence) (Fig 1). DTW correspond to the optimal path that matches the point sequences.

DTW is calculated with the following equation 4.

$$DTW\;(Q,P)=\min\left[\overset{k}{\underset{k=1}{\sum }}d(q_{ik},p_{ik})\right]$$

where d(*q*_*ik*_, *p*_*ik*_) is the Euclidean distance between two points in the Q and P series [43]. The result is in arbitrary units. Higher values indicate a larger deviation from the reference trajectory.

HD and DTW are complementary parameters since HD relates to a particular point of the trajectory (the greatest of all the distances, for the sub-task analyzed) while DTW considers the trajectory as a whole (the sum corresponding to the optimal path between the two trajectories, for the sub-task analyzed).

All parameters were calculated for the global TUG and for each sub-task using Matlab (Mathworks, Inc.).

Subjects also underwent a clinical examination as detailed in Table 3.

**Table 3:** Clinical examination.

Impairments and disabilities examined	Scale
Spasticity (quadriceps, rectus femoris, hamstring and triceps surae)	Modified Ashworth
Strength (hip, knee and ankle flexor and extensor)	Medical Research Council
Sensation and proprioception of lower limb	Nottingham Sensory Assessment
Activities of daily living	Barthel index
Walking independence	New Functional Ambulation Classification score
Balance	Berg Balance Scale
Balance confidence	Activities-specific Balance Confidence Scale

### Statistical analysis

Performance time, DTW and HD were calculated for each sub-task of the TUG (Go, Turn and Return) as well as the total trajectory. Trajectory length was computed for the total TUG trajectory. As the parameters were not all normally distributed, medians and quartile ranges are presented and non-parametric tests were used. Mann-Whitney tests were used to compare patients and healthy subjects, fallers and non-fallers and patients with right and left hemisphere stroke. A Bonferroni correction was used (since four repeated comparisons were carried out) with an adjusted p of 0.0125. A logistic regression was performed for each sub-task of the TUG to assess the additional variance of the dependent measure (stroke/no stroke) accounted for by DTW and HD above and beyond that accounted for by TUG time and nuisance variables (sex, age, body mass index). DTW and HD were added together in the regression model. Correlations between the BBS scores and trajectory parameters were tested with Spearman’s correlation for both the patients with stroke and healthy subjects, and for each sub-task (p < 0.05 was considered as significant). All analyses were performed using Statistica (version 7.1)

## Results

### Comparison of trajectory parameters between stroke patients and healthy subjects

**Fig 2:**
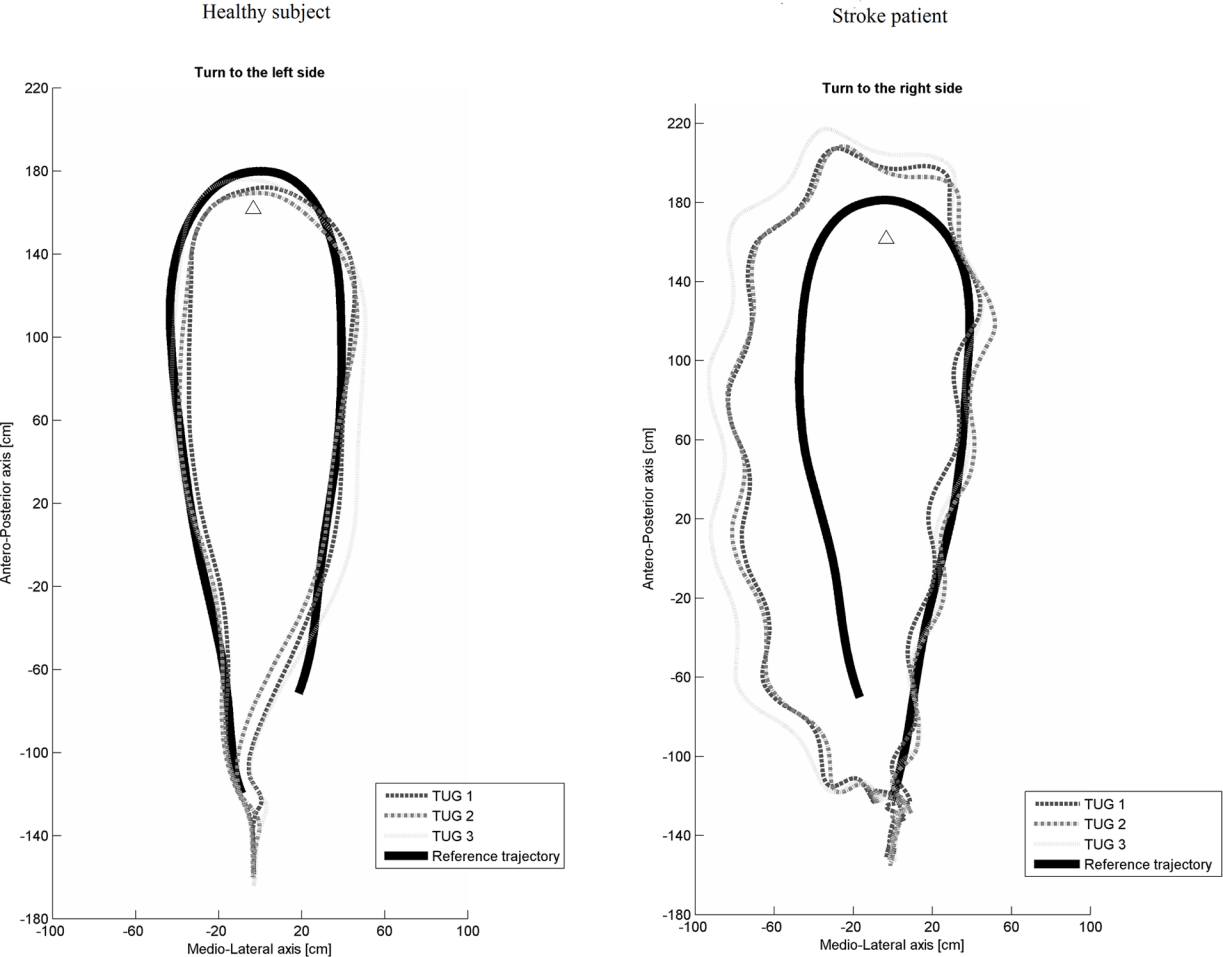
Trajectory of a healthy subject and a patient with stroke.

Results of the trajectory parameters are presented in Table 4. Fig 2 shows the trajectories of a patient with stroke and a healthy subject. Trajectory length, HD and DTW of the total TUG test were significantly greater in the stroke group (respectively p = 0.000001, p = 0.0001, p = 0.00004). HD and DTW were significantly greater in the stroke group during the Go (respectively p = 0.00002, p = 0.0009) and Turn (respectively p = 0.0002, p = 0.000001) sub-tasks. Both HD and DTW were greater in the patient group showing that, for a given sub-task, they deviated from the reference trajectory both at an isolated point (assessed with HD) and during the entire sub-task (assessed with DTW).

**Table 4:** Trajectory parameters [medians and interquartile ranges Q1;Q3] during the global trajectory and Go, Turn and Return sub-tasks of the TUG for both groups.

	Stroke group				Healthy group			
	Global	Go	Turn	Return	Global	Go	Turn	Return
HD (cm)	29.3 [21.9;33.3]	22.6 [17.1;28.5]	33.0 [25.4;42.2]	28.4 [22.1;37.4]	19.2 [17.5;23.9]*	15.1 [10.5;16.7]*	20.4 [18.7;26.8]*	22.8 [20.1;27.7]
DTW(arbitrary unit)	12983 [10576;19958]	4438 [3373;6139]	5238 [4344;7844]	5298 [3561;7745]	9023 [7522;10969]*	3017 [2187;3379]*	2252 [1875;2638]*	4783 [3631;6326]
Trajectory length (cm)	838,5 [817.7;864.5]	-	-.	-	750.1 [737.7;766.1]*	-	-	-
TUG performance (time in sec)	19.4 [15.9;21.5]	-	-	-	9.9 [9.5;11.5]*	-	-	-

HD Hausdorff distance

DTW Dynamic time warping

TUG Timed Up and Go

* significant difference between Stroke group and Healthy group for the sub-task (p<0.05)

### Additional information provided by the trajectory parameters

**Fig 3:**
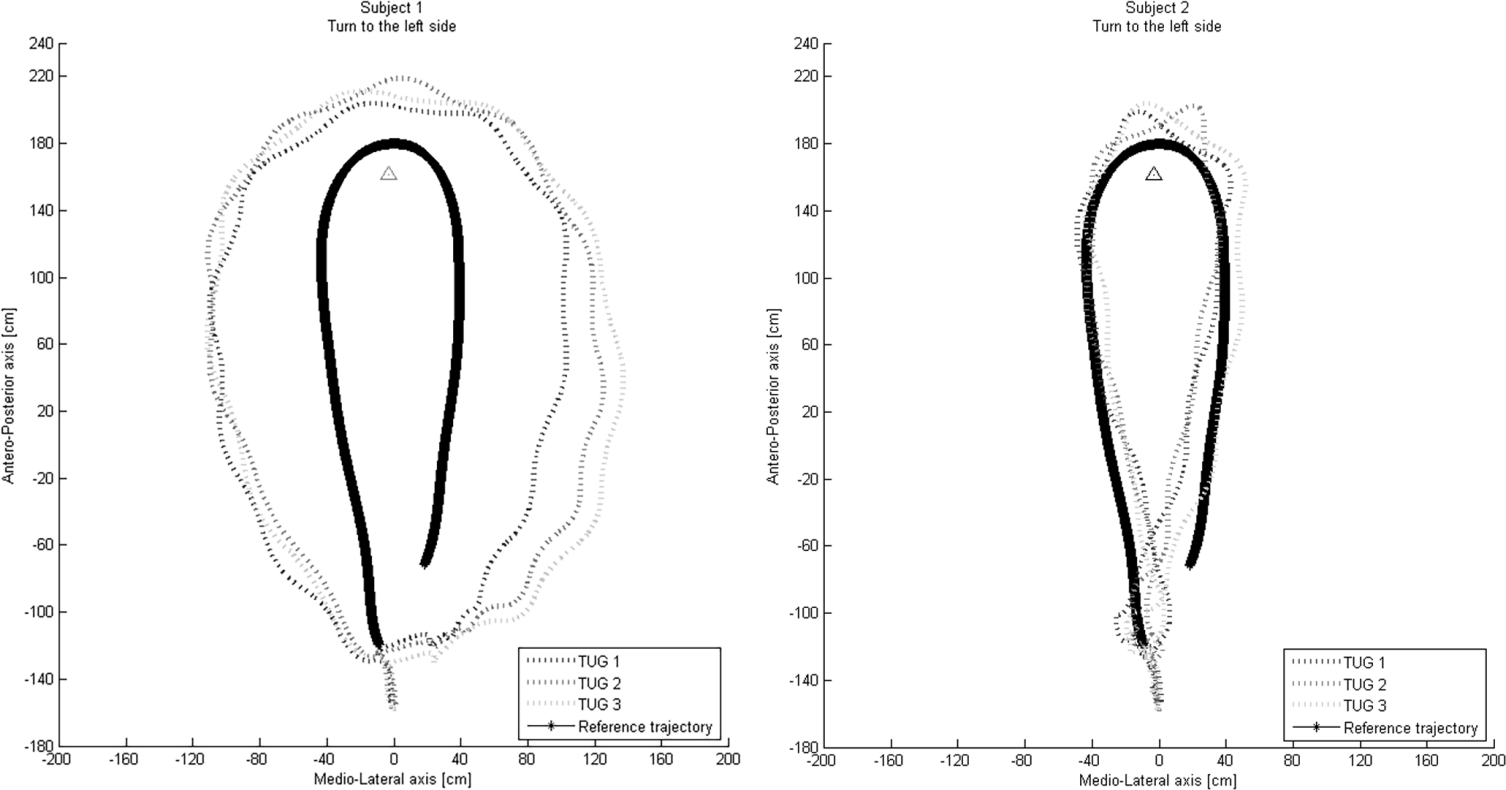
Trajectory of two characteristic patients with similar performance times (20.7 and 20.8s) but distinct trajectories.

The logistic regressions showed that the variance increased for the Go sub-task when the trajectory parameters were included. Indeed when all variables were included in the model the R^2^ was 0.56 and when the trajectory variables were not included (model with time and nuisance variables) the R^2^ was 0.39. The results of the predictive factors of the logistic regression for Go are presented in the appendix (S1 Table). For the Turn and Return sub-tasks, the trajectory parameters did not provide additional information (not selected in the multivariate model, p<0.05). Fig 3 presents the trajectory of two characteristic patients with similar performance times but distinct trajectories, to illustrate the additional information provided by the locomotor trajectory parameters.

### Correlation between trajectory parameters and BBS score

There was a significant negative correlation between BBS score and trajectory length, HD and DTW during the total TUG (r between -0.53 and -0.68, p<0.05,). BBS score was also significantly correlated with DTW during the Turn (r = -0.6, p<0.05) but not with HD during this sub-task. No correlations were found for Go and Return.

### Comparison of trajectory parameters between fallers and non-fallers

DTW was significantly greater for fallers (n = 8) than non-fallers (n = 21) for the Go sub-task only (p = 0.005), no differences were found for the Turn, Return or the total TUG. There were no significant differences between fallers and non-fallers for HD and trajectory length during the total TUG or each sub-task.

### Comparison of trajectory parameters between patients with right and left hemisphere stroke

There were no differences for the DTW and HD for the Go, the Turn, the Return, the total TUG or for the total trajectory length between patients with right (n = 17) and left (n = 12) hemisphere stroke (p>0.05).

## Discussion

To our knowledge, this study is the first to analyze locomotor trajectories during oriented-gait involving curved paths and obstacle circumvention in stroke patients. The aims were i) to analyze the locomotor trajectories of patients with stroke during the walking and turning sub-tasks of the TUG using HD and DTW, and to compare them with healthy subjects; ii) to determine whether trajectory parameters provide additional information to the conventional measure (performance time); iii) to compare the trajectory parameters of fallers and non-fallers with stroke and of patients with right and left hemisphere stroke and iv) to evaluate correlations between trajectory parameters and BBS scores.

The results showed that, compared to healthy subjects, stroke patients had significantly longer total trajectories and larger deviations from the reference trajectory during the oriented-gait to the cone (Go) and the turning (Turn) sub-tasks. Lamontagne et al (2010) recently also found different locomotor trajectories in stroke patients compared to healthy subjects during overground walking in an environment which provided optic flow [17].

The results of the present study suggest that stroke patients exhibit different locomotor trajectories depending on the requirements of the sub-task. Differences in trajectory parameters between the patients with stroke and the healthy subjects during the oriented gait to the cone and the turn sub-tasks suggest that the perception of a visual target, explicitly associated with a plan to circumnavigateit, impacted the gait trajectories of the patients with stroke for reasons that remain to be determined.

Furthermore, the results of this study suggest that the analysis of locomotor trajectories is an interesting approach to the analysis of gait in patients with stroke, providing additional information to that of the conventional timed performance of specific locomotor tasks. The assessment of trajectory parameters complements timed performance, providing a more complete understanding of locomotor tasks in patients with stroke. This is supported by the results of the logistic regression analysis. Further studies are needed to determine to what extent patients with similar performance times differ in locomotor trajectory, and the factors that influence these differences.

Longer and more deviated trajectories were significantly related to poor balance during the turn sub-task. Moreover, the trajectories of the faller group were significantly more deviated than those of the non-faller group during the oriented-gait to the cone (Go). The patients’ gait parameters differed significantly from those of the healthy subjects during the oriented gait to the cone and the Turn. These sub-tasks both challenge stability. In contrast, the Return appeared to be less challenging since there were no significant differences between the parameters of the patients and healthy subjects, or of the fallers and non-fallers. Thus the Go and Turn appear to be the most challenging sub-tasks of the TUG test. Hicheur et al (2007) also found that “complex” locomotor trajectories (with a large turn amplitude) induce greater deviations from the mean than “simple” trajectories (with a smaller amplitude turn) in healthy subjects [16]. In the present study, the HD and DTW values of the stroke group were both greater than the values of the healthy subjects during the Go and Turn sub-tasks, revealing that deviations from the trajectory occurred throughout these sub-tasks and not only at an isolated point. It is possible that these larger deviations of trajectories throughout the obstacle circumvention task and the preceding phase compensate for instability. Our results are in accordance with these obtained in other patient groups. Older adults also increase the spatial margin when walking through apertures in comparison with young subjects [44]. Similarly, MacLellan and Patla (2006) showed that the locomotor trajectories of healthy subjects are modified proactively and retroactively when walking on a foam mat compared to overground. They suggested that these modifications of the locomotor strategy probably minimize threats to stability [45]. Maintaining a consistent but minimum spatial margin between an obstacle and the self has been suggested as one of the dominant control parameters to maintain balance and avoid perturbation [46]. However, the hypothesis that trajectory deviations could compensate for instability cannot be affirmed by our results and further studies will be necessary to confirm or infirm this.

Finally, we expected to find differences in the trajectories of patients with right and left hemisphere stroke since right hemisphere stroke may alter the perception of body verticality [18]. However, our results showed that there were no differences, suggesting either that there were no significant differences between our two groups of participants in the subjective vertical (which we did not measure) or that alterations in the subjective vertical did not affect the locomotor trajectories during the TUG test in this sample of patients with moderate to good recovery. Nevertheless this assumption should be tempered since the distribution of patients with right and left hemisphere strokes was slightly asymmetrical (twelve patients with left stroke and seventeen with right stroke).

### Limits and perspectives

The patients included in this study had mild impairments; therefore caution must be taken regarding generalization of the results. The lack of difference between patients with right and left hemisphere stroke should also be interpreted with caution since we did not carry out a specific assessment of subjective vertical and cognitive functions relating to spatial perception (e.g. hemi-spatial neglect). Further studies designed to assess the influence of perception on trajectory would be interesting. The analysis of the trajectories of the faller patients was not our initial objective which explains why this sub-group was small. This limits the interpretation of the data for the discrimination of fallers and non-fallers, however these preliminary results suggest that the analysis of trajectory parameters may be a relevant approach to address this issue. Further studies specifically designed to fulfil this objective are nevertheless necessary. It would also be interesting to study whether locomotor trajectories are influenced by sensory perturbations in patients with stroke. Moreover locomotor trajectory analysis could be an interesting approach to assess the impact of medical treatment (such as botulinum toxin), surgical treatment or rehabilitation on “real-life gait” instead of conventional straight-line gait analysis.

## Conclusion

This study presents an innovative approach to the quantitative analysis of locomotor trajectories in patients with stroke during oriented-gait and obstacle circumvention, based on the widely used TUG test. This approach complements timed performance since it objectively quantifies locomotor trajectory and provides additional information regarding gait alterations in the presence of an obstacle. We evaluated parameters which quantified deviation from a reference trajectory and found that the trajectory of patients with stroke was more deviated than that of healthy subjects during the turn and the phase preceding the turn. No differences were found between patients with right and left hemisphere stroke. Comparison of faller and non-faller patients also showed that trajectory parameters differed during the phase preceding the turn. These results suggest that assessing the locomotor trajectory in addition to timed performance during complex locomotor tasks such as those assessed during the TUG test (i.e preparing to circumnavigate an obstacle and turning) might be relevant in patients with stroke and might also provide a basis for estimation of fall risk.

## Supporting Information

S1 Table Logistic regression for the Go sub-task of the TUG: predictive factors. Caption: 1* reference value; OR odds ratio, CI confidence interval. NS non-significant
(DOCX)
