# Supplementary material for: Locomotor Trajectories of Stroke Patients during Oriented Gait and Turning
Source: PLoS One. 2016 Feb 19;11(2):e0149757. doi: 10.1371/journal.pone.0149757 (PMC4760702; doi:10.1371/journal.pone.0149757)
Supplement: S1 Table — Caption: 1* reference value; OR odds ratio, CI confidence interval. NS non-significant (DOCX) [file pone.0149757.s001.docx]

Appendix S1 Table: Logistic regression for the Go sub-task of the TUG: predictive factors

|  | **Univariate model** |  |  | **Multivariate model** |  |  |
| --- | --- | --- | --- | --- | --- | --- |
| **Variables** | **OR [CI95%]** | **P value** | | **OR [CI95%]** | **P value** |  |
| **Nuisance variables** |  |  |  |  |  |  |
| Female gender | 0.48 [0.16 ; 1.43] | 0,17 |  | - | - | - |
| Age _ yr | 1.02 [0.97 ; 1.08] | NS |  | - | - | - |
| Body Mass Index | 1.22 [1.02 ; 1.45] | **0,03** |  | 1.30 [1.06 ; 1.60] | **0,01** |  |
| **Walking variables** |  |  |  |  |  |  |
| TUG time Go | >999.9 [<0.001 ; >999.9] | NS |  | - | - | - |
| DTW Go |  |  |  |  |  |  |
| < 2799 | 1* | - | - | 1* | - | - |
| 2799 - 3469 | 4.12 [0.65 ; 26.0] | **0,13** | **0,007** | 5.10 [0.61 ; 42.99] | 0,13 | **0,007** |
| 3469 - 4747 | 20.16 [2.80 ; 145.24] | **0,003** |  | 44.24 [4.14 ; 473.19] | **0,002** |  |
| > 4747 | 18.33 [2.52 ; 133.21] | **0,004** |  | 21.41 [2.22 ; 206.31] | **0,008** |  |
| HD Go |  |  |  |  |  |  |
| < 14 | 1* | - | - | 1* | - | - |
| 14 - 17 | 2.29 [0.42 ; 12.50] | **NS** | **0,007** | - | - | - |
| 17 - 25 | 8.25 [1.45 ; 46.86] | **0,02** |  | - | - | - |
| > 25 | 22.00 [3.08 ; 157.34] | **0,002** |  | - | - | - |

1* reference value; OR odds ratio, CI confidence interval.

NS non-significant
